# The contribution of verbal delayed recall to phonemic but not semantic fluency in aging individuals

**DOI:** 10.1093/oons/kvag007

**Published:** 2026-08-04

**Authors:** E Wehling, A J Lundervold, D Wollschlaeger

**Affiliations:** Department of Clinical and Biological Psychology, Alrek helseklynge, Årstadveien 17, University of Bergen, 5009 Bergen, Norway; Department of Neurology, Haukeland University Hospital Bergen, Jonas Lies vei 71, 5053 Bergen, Norway; Department of Clinical and Biological Psychology, Alrek helseklynge, Årstadveien 17, University of Bergen, 5009 Bergen, Norway; Institute of Medical Biostatistics, Epidemiology and Informatics, University Medical Center of the Johannes Gutenberg-University Mainz, Langenbeckstr. 1, 55131 Mainz, Germany

**Keywords:** phonemic, semantic, semantic knowledge, delayed recall, inhibition

## Abstract

**Objective:**

The cognitive abilities contributing to verbal fluency performance in aging individuals remain discussed. Besides executive functioning and processing speed, memory retrieval is necessary for successful verbal fluency performance. The latter has often been overlooked, although it may use similar retrieval mechanisms with verbal fluency performance. We aimed to investigate cognitive abilities contributing to phonemic and semantic fluency during the time course of the tasks.

**Methods:**

One hundred and thirty-two healthy aging individuals were assessed with measures of verbal fluency, processing speed, executive functioning, word knowledge and delayed recall. Correlation analyses assessed pairwise associations between variables. Verbal fluency performance was modeled using Poisson regression, accounting for repeated measures.

**Results:**

We observed significant correlations between verbal fluency and several cognitive measures and sex. Different cognitive predictors were found for phonemic and semantic fluency performance. Phonemic fluency was associated with word knowledge and inhibition while shifting ability was important for semantic fluency. Delayed recall was a significant predictor in the late interval during phonemic fluency.

**Conclusion:**

Our findings demonstrate that multiple cognitive abilities contribute to verbal fluency performance. More cognitive measures were associated with phonemic than semantic fluency, delayed recall being one of them. An increased understanding of contributing factors to fluency enables clinicians and researchers to meet individuals experiencing age-related changes in fluency performance.

## Introduction

Verbal fluency tests are commonly applied in clinical and research settings due to their sensitivity to brain impairment and their ease in administration (Lezak et al. 2012, Rabin et al. 2005). In the assessment of aging populations, fluency tests play an important role, since performance on these tests has been shown to predict the progression of neurodegenerative conditions such as Alzheimer’s disease, as well as the conversion from mild cognitive impairment to dementia (Cintoli et al. 2024, McDonnell et al. 2020). Commonly, an individual is asked to generate words starting with a specific initial letter (phonemic fluency) or belonging to a given category (semantic fluency) within a limited time interval (typically 1 min). These tasks involve several cognitive abilities including the immediate initiation of word generating, having a set of words available to choose from (word knowledge), the capability to retrieve words from memory and executive abilities to coordinate this process (monitoring of responses including inhibition of irrelevant responses, avoidance of repetition, and the flexibility to shift mental sets). From a neuropsychological perspective, the contributions of individual cognitive functions, age and diagnosis are still debated (Abwender et al. 2001, Gonzalez-Burgos et al. 2019, Henry and Crawford 2004a, 2004b, 2004c, Henry et al. 2004, Kljajevic 2026, Metternich et al. 2014, Stolwyk et al. 2015).

Understanding the cognitive functions and their contributions to verbal fluency is essential for clinicians to interpret performance patterns as well as to become able to detect pathological changes. This is particularly important in aging individuals as demographic change increases their number, and best practice guidelines to identify cognitive disorders such as Alzheimer’s and Parkinson’s disease include fluency assessment (Litvan et al. 2012, Paajanen et al. 2014).

Phonemic fluency has frequently been taken as an ability strongly tapping executive functioning (EF) since performance seems to require strategic search for words, monitoring of responses including inhibition of irrelevant response, avoiding repetitions, and the flexibility to shift mental sets (Amunts et al. 2020, Raboutet et al. 2010, Rende et al. 2002, Stolwyk et al. 2015, Troyer et al. 1997). Semantic fluency requires both executive and verbal/semantic abilities (VA) such as verbal knowledge and lexical retrieval (Bryan et al. 1997, Kave and Mashal 2012, Salthouse 1993, Troyer et al. 1997). The distinct contributions of EF and VA may explain that fewer words are generated in phonemic than in semantic fluency tasks within the same time frame (Lezak et al. 2012). It has been suggested that the search process in phonemic fluency is more demanding since it is uncommon in speech production and thus less automatic than semantic retrieval. Semantic fluency is purported to depend heavily on the access to and integrity of semantic stores (Rosen and Engle 1997). The initiation of responses may lead to further activation of related semantic neighbors and search in related semantic networks (Henry and Phillips 2006, Hirshorn and Thompson-Schill 2006, Katzev et al. 2013, Luo et al. 2010, Rende et al. 2002, Rosen and Engle 1997).

In aging individuals, performance patterns seem conflicting with regard to the EF and VA differentiation since greater decline is commonly reported in semantic fluency, although semantic knowledge is known to be stable or even improving with age (Crossley et al. 1997, Henry and Phillips 2006, Rodriguez-Aranda and Martinussen 2006, Van der Elst et al. 2006). Troyer et al. (1997) demonstrated that patterns of clustering (producing words within a semantic category) and switching (identifying and shifting between categories) change with advancing age. They proposed that age-related declines in semantic fluency are primarily driven by reduced switching ability, highlighting the role of executive functions. Other explanations stem from either a global approach towards age-related changes, assuming a general global decline affecting several cognitive domains or an approach suggesting specific changes in the domain of language processing.

The processing speed theory suggests that cognitive slowing impairs task performance via either time limitation (mental operations exceed the allotted timeframe) or simultaneity failure (necessary information is no longer available at later processing stages) (Salthouse 1996). Findings supporting this hypothesis reveal that age-related differences in specific cognitive measures (e.g. attention, memory), are reduced when controlling for the effect of processing speed (Salthouse 2001, 2017). Yet, as for other functions, processing speed does not explain changes alone. Regarding verbal fluency, the processing speed explanation leaves the greater decline in semantic fluency unexplained since processing speed accounts for both conditions. Thus, there is also indication of domain-specific slowing (Madden and Allen 2015).

A process specific explanation is the transmission deficit hypothesis, explaining the asymmetrical decline of language production versus comprehension in aging (Burke and Shafto 2004, Mackay and Burke 1990). Based on the Node-Structure Theory (Burke et al. 2000), there are nodes representing the sentential system (semantic and syntactic representations) at the top-level and nodes representing the phonological system (including phonemes and syllables) at the lower level. In this framework, higher-level nodes have many-to-one connections, resulting in robust activation because a lexical node can be excited via multiple propositions. Yet, top-down activation from the sentential level to the phonological system relies on one-to-one connections between a lexical representation and its phonological form. Since aging is assumed to weaken the strength of connections between nodes, the later stage of transmission has shown to be particularly vulnerable to aging (Mackay and Burke 1990). In phonemic fluency, retrieval may depend primarily on phonological representations, whereas semantic fluency requires linking conceptual representations to their phonological forms. Consequently, semantic fluency is more vulnerable to impairments in activation transmission (Burke and Shafto 2004, Kave and Mashal 2012). In a recent study, Kljajevic (2026) suggested that slower processing speed due to aging could affect all levels of word retrieval, including slower conceptual activation and lexical selection on the top level and on the phonological level causing slower coordination of phonological representations and motor programs. She addressed the importance of other cognitive functions like working memory, reasoning, verbal memory on forming representations in the model.

Studies investigating which cognitive functions contribute to fluency performance to fluency in aging, and why individuals differ in performance, have yielded conflicting results. Examining associations in 70–98-year-old individuals, one study showed that phonemic fluency was predicted by verbal intelligence and processing speed (Bryan et al. 1997). Stolwyk et al. (2015) found that verbal intelligence predicted phonemic fluency while none of the included variables (verbal intelligence, semantic retrieval, processing speed, working memory or inhibition) predicted semantic fluency in 65–87-year-old individuals. A study examining both phonemic and semantic fluency performance in 46–85 years olds (n = 224) showed that lexical access, processing speed and executive functions were the strongest contributors to both phonemic and semantic fluency tasks (Gonzalez-Burgos et al. 2019). These authors suggested that other cognitive functions susceptible to age-related changes influence the dynamic changes in performance and may serve to compensate for decline.

Beyond processing speed and executive functioning, memory is involved when retrieving words. So far, empirical evidence examining the contribution of verbal memory to fluency performance is still limited. Existing studies indicate that performance on delayed verbal recall predicts fluency in both young and older adults, for both phonemic and semantic fluency (Gonzalez-Burgos et al. 2019, Kave and Sapir-Yogev 2020, Kljajevic 2026, Ruff et al. 1997, Unsworth et al. 2011, Weiss et al. 2006). The association between delayed verbal recall and fluency tasks is of particular interest since both measures are commonly used in the assessment of older individuals and those where there is a suspicion of pathological cognitive decline (Weissberger et al. 2017).

Since the speed of producing words on both phonemic and semantic fluency changes significantly over the time course of the respective task, the importance of examining temporal performance patterns to further untangle the contribution of different functions during performance has been highlighted (Crowe 1998, Fernaeus and Almkvist 1998, Mayr and Kliegl 2000, Raboutet et al. 2010). Early in the tasks, word production primarily depends on access to easily available words, whereas later stages require a controlled strategic search in memory, a more effortful process (Crowe 1998, Fernaeus and Almkvist 1998, Mayr and Kliegl 2000, Raboutet et al. 2010, Rende et al. 2002, Rosen and Engle 1997). One of the few studies addressing the contributions of cognitive functions within the course of the tasks in aging individuals (normal aging, adults with Alzheimer’s (AD) or Parkinson’s disease (PD)), revealed that processing speed, inhibition and working memory were significant predictors for letter fluency, while processing speed and inhibition predicted semantic fluency (McDowd et al. 2011). The authors did not find a clear dissociation between the contributing cognitive functions throughout the course of the task. Yet, time courses seemed to be particularly sensitive in adults with AD.

In sum, verbal fluency has a hybrid character involving several cognitive functions whose contributions may vary across the time course of the tasks. To advance interpretation of performance patterns, it is relevant to untangle their contribution throughout the time course of the tasks. Delayed verbal recall is a relevant function to investigate since its age-related changes are well-established (Greene and Naveh-Benjamin 2023, Lundervold et al. 2014) but its association with verbal fluency has rarely been addressed. We aimed to examine the contributions of demographic measures and cognitive performance scores including delayed verbal recall on verbal fluency tasks in community-dwelling aging individuals. We hypothesize overall better performance on semantic than phonemic fluency with a drop in performance throughout the tasks, most markedly in semantic fluency. Moreover, we expect significant associations between word knowledge, executive function, and processing speed and phonemic fluency whereas semantic fluency is expected to be associated with word knowledge and executive function. Delayed recall should be significantly associated with performance on both fluency tasks.

## Methods

Participants were recruited for a larger study on cognitive aging (Wehling et al. 2010, 2011). Participants aged 45–70 years were included. Participants with acquired neurological and psychiatric disorders or head trauma were excluded. Of 163 participants originally included, n = 31 participants were excluded in this study since they were not assessed with the verbal fluency tasks due to time-constraints during their visits. In total, this study included n = 132 community-dwelling aging individuals (see Table 1 for sample characteristics). To control for potential mild cognitive impairment, we verified that none of the participants scored 1.5 SD below age-matched normative data on the CVLT delayed recall, a commonly used cutoff for mild cognitive impairment (Petersen 2004). All subjects provided informed consent according to the Declaration of Helsinki. The project was approved by the Regional Committee for Research Ethics of Western Norway.

**Table 1:** Sample charcteristics and performance on neuropsychological measures.

	Participants n = 132; M (SD)	Neuropsychological performance T-score M (SD)
Sex (male/female)	44/89	
Age (years)	60.5 (7.6)	
Education (years)	13.6 (3.2)	
Verbal knowledge (Vocabulary), sum	64.6 (7.7)^*^	57.6 (8.7)
Visual reasoning (Matrices), sum	23.9 (5.6)^*^	58.1 (8.7)
Inhibition (Color Word Interference Test), seconds	57.3 (13.7)^*^	55.4 (2.5)
Cognitive flexibility (Trail Making Test, B), seconds	76.7 (26.9)^*^	51.4 (8.3)
Processing Speed (Digit Symbol Test), seconds	49.3 (9.5)^*^	45.4 (8.1)
Memory (Delayed recall, CVLT), sum	12.1 (2.9)^*^	55.1 (1)

^^*^^Raw scores

### Cognitive assessment

Participants were assessed with the tests described below. To provide an indication of performance levels, standardized scores were computed for each measure according to normative data from the respective test manuals (Table 1).

### Verbal fluency tasks

All participants were asked to perform the Verbal fluency tasks from the Delis-Kaplan Executive Functioning System (Delis et al. 2001). This version of verbal fluency tests comprises three conditions of which two are included in this study. In the *Phonemic fluency task*, the participant was asked to generate words beginning with a certain letter (F-A-S) within 60 sec per letter. Names (places and people) and numbers were not allowed. In the *Semantic fluency task*, the participants were asked to generate words belonging to two semantic categories (animals, boys’ names) with 60 sec per category. They were instructed to avoid repetitions in any of the tasks. All participants first performed the phonemic fluency subtests, followed by the semantic fluency subtests according to the manual. All words (correct answers, repetitions and set loss errors) were recorded in writing using designated sheets within 15 second intervals. Words generated while crossing the border between intervals were attributed to the earlier interval. The scores included were the total number of correct words, total number of errors (repetition and set loss errors) and number of words generated within each 15 second interval.

### Executive functioning – Inhibition and shifting

The third subtest of the CWIT from the D-KEFS battery (Delis et al. 2001) was include in the analyses as a measure of inhibition. In this subtest, one of three color words (‘blue’, ‘green’ and ‘red’) printed in an incongruent ink color are presented. Over 50 repetitions, the participant’s is asked to name the ink color. This task requires inhibition of the automatized response of word reading. Scores are the time elapsed to complete the task.

As a measure of shifting and cognitive flexibility, the B-condition of the Trail Making Test (TMT) (Reitan and Wolfson 1985, Tombaugh 2004) was included. Here, the participant must alternately connect numbers and letters on a sheet of paper (i.e. 1-A-2-B-3-C etc.). The amount of time required to complete the task was the score included in the analysis. Although the Trail Making Test Part B (TMT-B) is commonly viewed as a measure of cognitive flexibility, some authors argue that flexibility may not be the primary contributing factor (Lezak et al. 2012). Since for both the CWIT and the TMT-B tests scores represent the time elapsed to complete the task, higher numbers indicate lower performance on the task.

### Processing speed

The Digit Symbol Test, a subtest from the Wechsler Adult Intelligence Scale-R (WAIS-R; Wechsler 1981), is a test of psychomotor performance which is fairly unaffected by intellectual ability, memory or learning. Earlier findings reveal that approximately half of the total score value of the Digit Symbol Test is based on copy speed (Storandt 1976). The test consists of rows with black squares each containing a randomly assigned number. Above the rows, a key is presented paring each number with a symbol. After a practice trial, the participant is required to fill the blank spaces with the designated symbols for 120 seconds. The score is the number of correctly filled squares.

### Verbal knowledge

The Vocabulary subtest from the Wechsler Abbreviated Scale of Intelligence (WASI; Wechsler 1999) assesses verbal concept formation and verbal reasoning. It consists of 40 words, listed in order of difficulty. The participant is asked to define words, and responses are scored as 2-, 1- or 0-point definitions according to the manual. The sum of achieved points was included in the analysis.

### Delayed recall

The California Verbal Learning Test (CVLT-II) (Delis et al. 2000) is a word learning task. Initially, 16 words (List A), drawn from four semantic categories, are presented five times. After each presentation, the participant is asked to repeat as many words as possible. Immediately after the fifth trial, a new list (List B) is presented, and the participant is asked to recall as many words as possible from that list (B). Immediately after recalling items from list B, the participant is asked to recall the words of List A, first without cues (free) which is followed by a cued recall condition. After 20 minutes delay, the participant is again asked to recall the words of List A without and with cues (free and cued delayed recall, respectively). Finally, a yes-no recognition test is presented, including the 16 items from List A, the 16 from List B and 16 random distractor items. In the present study we included the sum of words recalled without cues after the 20-min delay (CVLT – Free delayed recall) as a measure of delayed recall function.

### Data analysis

Descriptive analyses were run to describe characteristics of the participants and their performance on all included measures. Fluency performance scores were descriptively summarized as the total number of produced words overall. For phonemic fluency, words were summed over all three one-minute runs – one for each letter F, A, S. For category fluency, words were summed over the two one-minute runs, one for animals – and one for boys’ names. Furthermore, we calculated the average number of words produced per minute (WPM) overall as well as for each of the four 15 sec intervals. For phonemic fluency and the first 15 sec interval, we took the sum of produced words over the first 15 sec in each of the three one-minute runs, divided the sum by 45 and multiplied it by 60 sec to convert to WPM. For category fluency, the analogous sum over the first 15 sec in each of the two one-minute runs was divided by 30 sec and multiplies by 60. The average WPM rates for the remaining intervals were calculated analogously. The relationship between performance in semantic fluency vs. letter fluency was described as the ratio between the respective WPM rates.

Pearson correlations were calculated to examine the linear association of overall verbal fluency performance with demographic characteristics (age, sex, and education) and the cognitive performance measures (processing speed, inhibition, shifting, word knowledge, delayed recall).

To assess the association of verbal fluency performance with demographic and neuropsychological performance scores over the time course of the task, we used Poisson regression models – one model for each fluency task as a dependent variable. In each model, each participant contributed four performance values – one for each 15 sec intervals of the one-minute task. For phonemic fluency, a participant’s performance value for the first 15 sec interval was the sum of produced words over the first 15 sec in each of the three one-minute runs. For category fluency, the corresponding performance value was the sum of produced words over the first 15 sec in each of the two one-minute runs. The performance values for the remaining intervals were calculated analogously.

Both models included the same set of predictors, including sex as well as neuropsychological performance scores (inhibition, shifting, delayed recall, processing speed, and word knowledge). Furthermore, the time during the task was included as a continuous variable, taking on the interval mid-points of 7.5/60 min, 22.5/60 min, 37.5/60 min, and 52.5/60 min as values. The regression models included pairwise interaction terms of performance measures with time to be able to take into account that the contribution of cognitive abilities to verbal fluency may change over the time course of the task. Log-time served as the offset to model fluency performance as rates of produced words per minute (phonemic fluency: log(45/60), semantic fluency: log(30/60)).

The predictors were chosen based on theoretical considerations except for vocabulary. Since education and vocabulary scores were strongly correlated, only vocabulary was included. This was based on the notion that education is often used as a proxy for verbal intelligence. However, since we had vocabulary as a direct measure, we chose to discard education. Age was not included in the regression models since age effects on verbal fluency performance are likely mediated by the performance in specific cognitive domains. Since each participant contributed four measurements of the dependent variable in each regression model, the method of generalized estimating equations was used with a robust sandwich estimator of the covariance matrix that also accounts for possible overdispersion (Højsgaard et al. 2006).

Multiple imputation by chained equations was used to deal with missing data among the performance measures (Word knowledge n = 5, Inhibition n = 3, Processing speed n = 1, Shifting n = 15) in the regression models (Van Buuren 2011). Missing values were imputed using predictive mean matching, taking sex, age, education, and available neuropsychological performance scores as covariates. The respective regression results from 20 imputed data sets were pooled using Rubin’s rules. The multiple imputation approach was based on the notion that when data is missing at random, multiple imputations can reduce selection bias as compared to a complete case analysis, in particular with respect to error variance estimates. A complete case analysis was carried out as a sensitivity analysis. Since interaction terms may render results from regression models difficult to understand intuitively, we used the method of estimated marginal means (Lenth and Piaskowski 2026) to illustrate model predictions. Model predictions for women were calculated separately for the observed range of predictor values for each cognitive performance measure at two different time points in the course of the verbal fluency task while keeping all other covariates at their sample mean. Confidence intervals for marginal means were calculated using the delta method. Significance level was set to p < 0.05 for all analyses if not indicated specifically. The statistical environment R version 4.5.1 was used for all analyses (R 2025).

## Results

### Characteristics of the sample

Demographic characteristics and cognitive performance scores are shown in Table 1. There were more female participants in the sample (67%; *n* = 89), age ranged from 45–77 (M 60.5 SD 7.6) years, and education from 8–20 (M 13.6 SD 3.6) years. Age and education did not differ significantly between men and women (p > 0.2). Performance scores on neuropsychological tests indicated that overall performance was somewhat above average based on peer normative data.

### Verbal fluency performance

**Figure 1:**
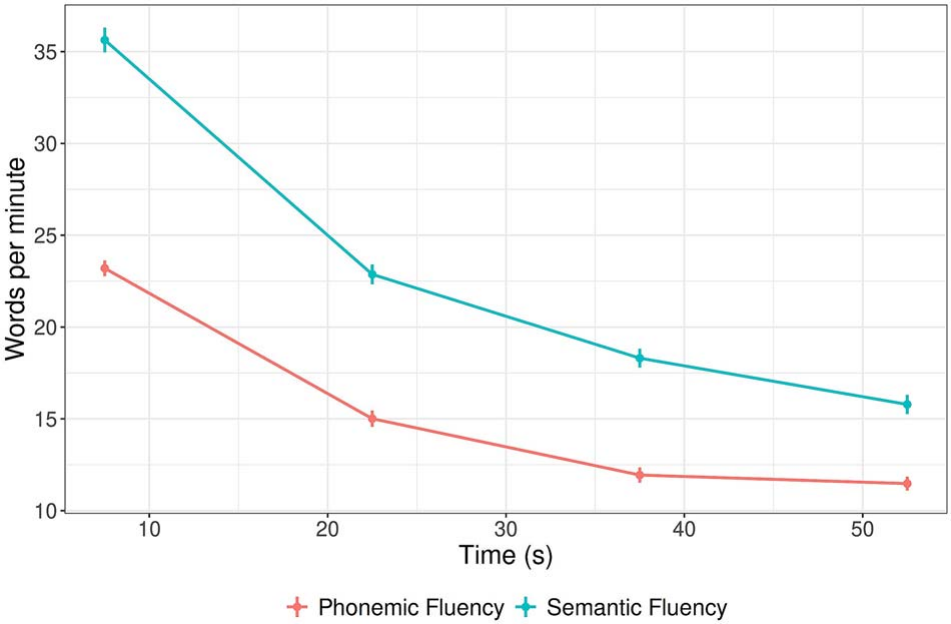
Observed average number of produced words per minute in phonemic and semantic fluency tasks expressed as means together with standard errors of the mean for each 15 s interval over the course of the respective one-minute tasks.

Mean and SD values for verbal fluency performance based on total scores and average number of words per 15 sec intervals are shown in Table 2. Participants produced fewer words on phonemic than semantic fluency tasks. The average discrepancy between the two tasks, calculated as a ratio (semantic:letter fluency) was 1.56 (*range* 0.84–2.77), indicating that for 16 words produced for the semantic fluency task, on average about 10 were generated in the phonemic fluency task in the same time period. Two participants produced more words in phonemic than semantic fluency. Few mistakes occurred on either task, on average *M* = 1.2 (*SD* 1.4; range 0–7) in phonemic fluency and *M* = 0.7 (*SD* 1.0, range 0–5 in semantic fluency). Fifty-six participants (42%) and 72 participants (55%) did not make any mistake on phonemic and semantic fluency, respectively. The time course of word production is illustrated in Fig. 1 showing the observed average number of produced words per minute in semantic and phonemic fluency tasks for each 15 s interval.

**Table 2:** Total number of words and average number of words per minute (WPM) on phonemic and semantic fluency tasks. Words were summed over the whole time period (1 minute each for 3 letters in phonemic fluency, 1 minute each for 2 categories in semantic fluency). Average WPM was calculated overall as well as for each of the four 15 s intervals of the respective tasks. Means (*M*) and standard deviations (*SD*).

	Phonemic fluency Raw scores *M (SD)*	Semantic fluency Raw scores *M (SD)*
Total words	46.2 (11.5)	46.3 (8.9)
Overall, average WPM	15.4 (3.8)	23.1 (4.4)
First interval, average WPM	5.8 (1.3)	8.9 (2)
Second interval, average WPM	3.7 (1.3)	5.7 (1.7)
Third interval, average WPM	3.0 (1.2)	4.5 (1.5)
Fourth interval, average WPM	2.9 (1.1)	3.9 (1.6)
Total perseveration errors	0.1 (0.6)	0.1 (0.3)
Total repetition errors	1.2 (1.4)	0.7 (0.9)

### Correlation between demographic characteristics, cognitive performance scores and verbal fluency tasks

Correlation analyses indicated that the fluency tasks correlated significantly with each other (*r* 0.45, *p* < 0.01) as well as with demographic (sex) and cognitive performance scores (Table 3). Performance on the individual fluency tasks did not correlate significantly with age but with sex. Performance scores on phonemic fluency correlated strongly with verbal knowledge (Vocabulary), Inhibition (Color Word Interference Test) and memory (Delayed recall, CVLT II) (all *p* < 0.01) as well as with processing speed (Digit Symbol Test) and shifting (Trail Making Test B) (all *p* < 0.05). Semantic fluency correlated significantly with memory (Delayed recall, CVLT II), processing speed (Digit Symbol Test), Shifting (Trail Making Test B), verbal knowledge (Vocabulary) (all *p* < 0.01), and Inhibition (*p* < 0.05).

**Table 3:** Pearson correlations between fluency tasks and demographic characteristics and neuropsychological performance scores (significant correlations *p* < 0.05 in bold).

	Age	Sex	Education	Phonemic fl.	Semantic fl	Word knowledge	Processing speed	Inhibition	Shifting	Memory
Age	-									
Sex	0.11	-								
Education	0.15	0.04	-							
Phonemic fluency	−0.06	**0.26**	**0.35**	-						
Semantic fluency	−0.08	**0.36**	0.15	**0.45**	-					
Word knowledge (Vocabulary)	−0.07	0.04	**0.49**	**0.32**	**0.27**	-				
Processing speed (Digit Symbol Test)	**−0.43**	**0.24**	**0.32**	**0.22**	**0.40**	**0.21**	-			
Inhibition (Color Word Interference Test, cond. 3)	**0.33**	−0.15	**−0.27**	**−0.29**	**−0.21**	**−0.19**	**−0.49**	-		
Shifting (Trail Making Test B)	**0.41**	−0.04	**−0.26**	**−0.22**	**−0.31**	**0.23**	**−0.46**	**0.39**	-	
Memory (CVLT delayed recall)	**−0.24**	**0.41**	0.12	**0.26**	**0.40**	**0.33**	**0.35**	**−0.20**	−0.14	-

^^*^^ *p* < 0.05

### Predictors of verbal fluency performance

Poisson regression models for both fluency tasks included sex and cognitive performance measures as predictors (Table 4). Model diagnostics and collinearity analysis identified no patterns requiring adjustments to the models (electronic supplement Table 1 and 2).

**Table 4:** Results of Poisson regression models (estimated rate ratios, *RR*) predicting phonemic (a) and semantic fluency (b) performance with 95% confidence intervals (*CI*) and *p*-values.

a.	*RR*	*CI 95%*	*p*	b.	*IRR*	*CI 95%*	*p*
Intercept	19.067	[11.298–32.177]		Intercept	30.464	[18.224–50.926]	
Sex, female	1.107	[1.023–1.198]	**0.012**	Sex, female	1.099	[1.031–1.173]	0.004
Vocabulary	1.007	[1.002–1.012]	**0.010**	Vocabulary	1.003	[0.998–1.008]	0.176
Delayed recall	0.989	[0.974–1.005]	0.183	Delayed recall	0.999	[0.985–1.015]	0.969
Inhibition	0.997	[0.993–1.000]	0.051	Inhibition	1.000	[0.997–1.004]	0.826
Processing speed	1.001	[0.995–1.007]	0.649	Processing speed	1.002	[0.997–1.007]	0.470
Shifting	1.000	[0.998–1.002]	0.805	Shifting	0.998	[0.996–0.999]	**0.037**
Time	0.994	[0.989–0.999]	**0.028**	Time	0.985	[0.977–0.993]	**< 0.001**
Delayed recall ^*^Time	1.000	[1.000–1.000]	**0.008**	Delayed recall ^*^Time	1.000	[0.999–1.000]	0.095
Shifting ^*^ Time	0.999	[0.999–1.000]	0.296	Shifting ^*^ Time	1.000	[0.999–1.003]	0.494
Inhibition ^*^ Time	1.000	[0.999–1.000]	0.918	Inhibition ^*^ Time	1.000	[0.999–1.000]	0.989
Processing Speed ^*^Time	0.999	[0.999–1.000]	0.281	Processing Speed ^*^Time	1.000	[0.999–1.000]	0.484

In the model for phonemic fluency, sex was positively associated with performance. Being female was associated with a 11% higher performance score compared to being male (Rate Ratio [*RR*] 1.107; 95% *CI* [1.023–1.198], *p* < 0.02). Furthermore, word knowledge was positively associated with higher phonemic fluency performance with a 0.7% higher predicted rate for each raw score point (*RR* 1.007; 95% *CI* [1.002–1.012], *p* < 0.02). Longer processing time in the inhibition task was negatively associated with phonemic fluency performance (*RR* 0.997, 95% *CI* [0.993–1.000], *p* = 0.05). This indicated that less time to complete an inhibition task (better performance) was associated with better fluency performance. The model revealed further that predicted performance declined significantly over the time course of the task (*RR* 0.983, 95% *CI* [0.967–0.0.998], *p* < 0.03). We found a significant interaction term indicating that delayed verbal recall was more strongly associated with phonemic fluency performance later in the course of the task (*p* < 0.01). For illustration, an additional 5 words in the delayed recall task were associated with 35% predicted increase in phonemic fluency performance (RR = 1.35, 95% CI [1.07–1.69]) at 10 sec, and with 91% predicted increase at 20 sec (RR = 1.91, 95% CI [1.81–3.09]).

**Figure 2:**
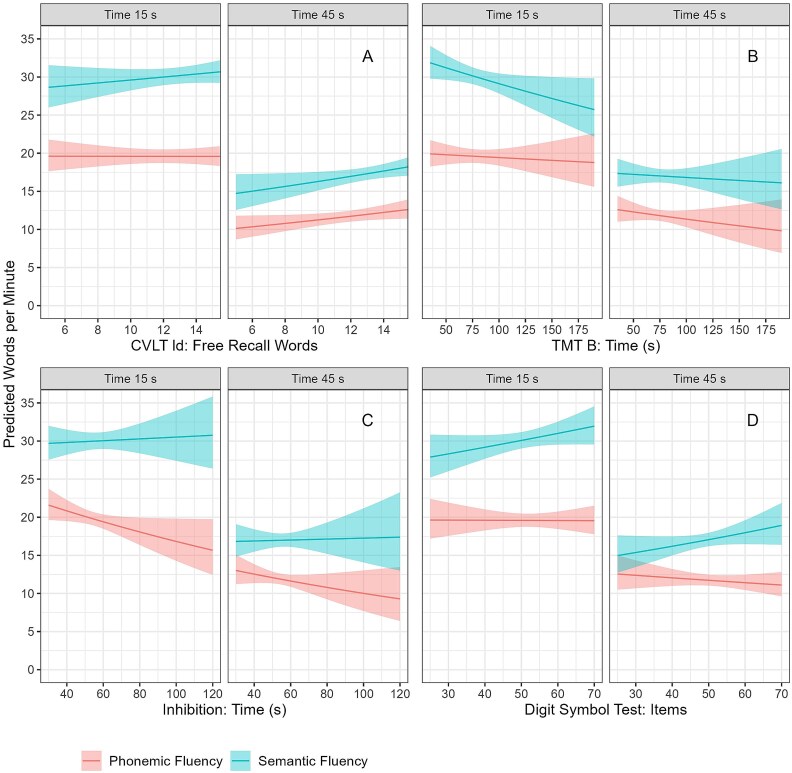
Predicted number of words per minute in phonemic and semantic fluency tasks derived from taskspecific Poisson regression models with a shared set of covariates including sex, word knowledge as well as cognitive performance in memory (CVLT, panel a), shifting (TMT B, panel B), inhibition (panel C), processing speed (digit symbol test, panel D), and time during the course of the respective tasks. Predictions were derived as estimated marginal means for females, for two time points during the task, and for the observed range of values for a single cognitive performance measure while setting all remaining covariates to their sample mean.

In the model for semantic fluency, sex was positively associated with performance with women having a 10% higher predicted performance than men (*RR* 1.100; 95% *CI* [1.031–1.173], *p* < 0.01). The shifting score was negatively associated with semantic fluency performance (*RR* 0.998, 95% *CI* [0.996–0.999], *p* < 0.04). This indicated that less time to complete the shifting task (better performance) was associated with better fluency performance. In this model, we found that predicted performance declined over the time course of the task (*RR* 0.970, 95% *CI* [0.958–0.987], *p* < 0.01). The interaction term suggested that delayed recall was more strongly associated with semantic fluency performance later during the course of the task without reaching statistical significance (*p* = 0.09). Here, an additional 5 words in the delayed recall task were associated with a 25% predicted increase in category fluency performance at 10 sec (RR = 1.25, 95% CI [0.999–1.577]), and with a 58% predicted increase at 20 sec (RR = 1.58, 95% CI [0.96–2.58]). Estimated marginal means visualizing the associations of predictors with fluency performance for two time points are illustrated in Fig. 2. Results from the complete case sensitivity analysis did not differ meaningfully from those of the primary analysis (electronic supplement Figure 1–4).

## Discussion

The aim of the study was to investigate the contributions of cognitive abilities to phonemic and semantic fluency and the potential changes of their impact during the time course of verbal fluency tasks. The participants showed higher performance in semantic than phonemic fluency. Sex and several cognitive functions including verbal knowledge, processing speed, executive functioning and delayed recall correlated with the verbal fluency performance. Regression analyses revealed a performance drop in both fluency tasks over their respective time courses, with different predictors contributing to the overall performance. The association of some predictors with fluency performance changed over the time course of the respective task.

Bivariate correlations showed no association of verbal fluency performance with age, in contrast to previous studies (Bryan et al. 1997; Henry and Phillips 2006; Rodriguez-Aranda and Martinussen 2006 but see also Sauzeon et al. 2010; Shao et al. 2014). Varying findings may be due to the included age-ranges in the studies. We focused specifically on aging individuals to be able to interpret the performance patterns of this demographically growing group (Shao et al. 2014, Stolwyk et al. 2015). Age correlated with all cognitive measures except word knowledge and may therefore have affected our results indirectly. Being female was associated with consistently better performance on both verbal fluency tasks. There is still a debate regarding sex-differences on fluency tasks. In their large meta-analysis, Hirnstein et al. 2023 concluded that there is a small female advantage in phonemic fluency while sex-differences in semantic fluency seemed category dependent. The authors suggestion that sex should be considered when fluency tests are used in clinical/diagnostic settings is supported by our data.

Our results demonstrated lower performance on phonemic than semantic fluency, which corroborates earlier studies (Gordon et al. 2018, Shao et al. 2014). Proposed explanations for these findings include the order of the tasks (tiredness). Alternatively, increased demands on attention and executive capacities in phonemic fluency. The former aspect was not supported by studies showing similar patterns despite counterbalanced order of tasks or assessment at different time points (Sauzeon et al. 2010, Vaughan et al. 2016, Woods et al. 2016). We found that the relative decrease in performance over time was slightly larger in semantic than phonemic fluency (68 vs 64% over one minute). Other authors reported lager decline in semantic performance (Gordon et al. 2018). Our results are based on performance as a continuous variable impeding direct comparison with those earlier reports. Yet, for clinicians it seems interesting to acknowledge how much decline can be expected during the course of the task and how much various cognitive variables contribute to performance. This will be discussed below.

Phonemic fluency performance correlated with performance scores from tests of processing speed, shifting, word knowledge, inhibition and delayed recall. The regression analyses revealed that word knowledge significantly contributed to fluency performance with a suggested contribution from inhibition (*p* = 0.05). Better word knowledge was positively associated with phonemic fluency performance, as was inhibition performance as indicated by faster completion of the inhibition task. Previous studies including inhibition as a predictor of phonemic fluency reveal ambiguous results. Analyses including overall fluency measures (Amunts et al. 2020, Unsworth et al. 2011) as well as those focusing on phonemic fluency underline inhibition as a significant predictor for fluency performance (McDowd et al. 2011) (but see also (Stolwyk et al. 2015). The suggestion is that inhibition serves to suppress inappropriate responses. Based on our data we cannot conclude if this explanation applies to our findings. Yet, it has been shown that semantic activation occurs even in purely phonological tasks (Vonberg et al. 2014). Thus, activated words may have to be inhibited when performing phonemic fluency tasks. Earlier ambiguous findings regarding the contribution of inhibition may be due to the diversity of measures applied which tap different types of inhibition (Shao et al. 2014, Shao et al. 2013). The task used in our study measures cognitive inhibitions, that is resisting extraneous thoughts/memories which seems to be similar to resisting to produce unmeant words (Diamond 2013).

The importance of word knowledge is manifested in that phonemic fluency requires retrieval of words from mental lexicon. Several authors suggest that individuals draw intensively on word knowledge (Hughes and Bryan 2002, Ruff et al. 1997) and may thus compensate for age-related reduction in other functions such as processing speed or executive functions in the later intervals (Salthouse 1993, Sauzeon et al. 2010, Stolwyk et al. 2015). A notable finding was therefore that delayed verbal recall was strongly associated with phonemic fluency performance later during the task. To begin with, these results stress the importance of time course analyses. Furthermore, is seems to support suggestions that that both fluency and delayed recall require controlled strategic search in long-term memory (Kave and Sapir-Yogev 2020). Although the distinct mechanisms remain unclear, one explanation is that search becomes more controlled and needs additional resources when easily available words (frequent, typical wors) have been produced, in that is later in the task. However, this reasoning has so far mostly been applied to semantic fluency (Shao et al. 2014) and needs more examination in phonemic fluency. Another explanation comes from a study including different measures of delayed recall (Gonzalez-Burgos et al. 2019) reporting that one measure (story recall) stopped contributing to verbal fluency after the age of 70, whereas another measure of delayed recall (list learning) started to contribute at that age. These authors proposed that varying contributions of measure may reflect compensatory mechanisms in order to maintain a consistent performance level in aging. Based on ours and those earlier findings we suggest that the administered tasks each engage multiple cognitive functions. The delayed recall task included in our study (list learning) is known to blend retrieval, executive function (retrieval strategy, semantic organization), language ability and attention where participants must actively structure information for effective storage and retrieval. This highlights the involvement of executive functions. Story memory tasks involve less active semantic processing and may thus lead to other results (Tremont et al. 2000). In sum, our findings support the notion that phonemic fluency is a measure of executive functioning, while the importance of both word knowledge and delayed verbal recall is emphasized.

Semantic fluency is recognized as a measure of executive and semantic retrieval abilities. In our study, it correlated with measures of inhibition, word knowledge, shifting, processing speed, and delayed verbal recall. The regression model indicated that shifting performance was a significant predictor as reported earlier (Jewsbury and Bowden 2017, Rodriguez-Aranda et al. 2006, Troyer et al. 1997). Although it seems plausible that the ability to shift is necessary to shift to new subcategories, our data does not allow us to differentiate the kind of shifting that may have occurred, i.e. the ability to disengage from a strategy or a category or the ability to initiate the search for a new strategy. Moreover, performance has shown to vary depending on the type of category (e.g. it may be easier to recall fruits from imagining the fruit shelf in one’s habitual supermarket vs. generating fluids) (Greenberg et al. 2009, McDowd et al. 2011). In contrast to our hypothesis, delayed verbal recall was not a significant predictor for semantic fluency although there may be some association later during the task (*p* = 0.09). This is in contrast to earlier findings in aging individuals (Gonzalez-Burgos et al. 2019, Kave and Sapir-Yogev 2020). As discussed earlier, different measures with underlying cognitive mechanisms may have contributed to these results (Coutinho et al. 2015, Tremont et al. 2000). Neither processing speed nor word knowledge were significant predictors for semantic fluency in our study, consistent with earlier work (Stolwyk et al. 2015).

Considering the findings and the contribution of the present study, our data does not enable us to draw conclusions about the strategies used in word retrieval in the fluency conditions. Yet, the findings demonstrate that varying cognitive functions are involved in fluency performance. Moreover, this is another study highlighting the complexity of a seemingly simple task (Kljajevic 2026, Ruff et al. 1997, Unsworth et al. 2011), not least with regard to the observed changes over time. The findings stress that although performance on fluency tasks may reflect underlying brain dysfunction, clinicians should be aware of the contribution from other cognitive functions. The findings highlight further that the primary focus on total correct scores should be reconsidered, with more emphasis on response slopes over time. Other measures, like list learning tasks, provide clinicians with profiles and measures for patient populations. Although existing, such measures have not been extensively examined for fluency tasks. Subjective complaints about word retrieval difficulties are documented in aging individual and a number of clinical populations (Brandstadter et al. 2020, Hedman et al. 2022, Ottarsdottir et al. 2024) highlighting to need to gain more knowledge on word retrieval and verbal fluency.

The present study has several strengths and limitations. Regarding limitations, the cross-sectional design precludes causal interferences and may introduce cohort effects that limit the generalizability of our findings. However, cross-sectional data retain value in this context since longitudinal studies may suffer from practice effects. Another limitation concerns the semantic fluency tasks from the D-KEFS which uses a composite measure for two categories (animals and boys names). Although both broad categories, participants may have applied different strategies during performance. We are aware that there are several other fluency measures that may have required more effort. This seems aimable in future studies examining fluency performance in aging individuals to further untangle contributing factors. Third, performance scores can vary across countries and cultural contexts (Pekkala et al. 2009), which may have influenced word output in the present sample. Finally, we included only one measure per cognitive domain while a higher number could have given more robust measures of cognitive functioning.

Nonetheless, our study has notable strength. A key one is the inclusion of both phonemic and semantic fluency tasks for all participants, allowing direct comparison of the same predictors across tasks in the same sample. Additionally, the measures used here are widely employed in clinical practice which enhances the practical relevance of the findings given ongoing demographic changes in aging populations. While single test-measures may yield less stable estimates than composite measures, they also shorten assessment sessions, likely improving participant compliance, and allow the unique contribution of individuals measures to be examined without conflation across tests.

## Conclusion

Including both phonemic and semantic fluency, the current study reveals that word knowledge, inhibition and delayed recall contribute to phonemic fluency performance while shifting ability contributed to semantic fluency performance in a group of aging individuals. These findings contribute to novel insight by examining whether cognitive measures interact with performance across different parts of the test, which was shown for delayed recall in phonemic fluency. A better understanding of fluency measures is essential for clinicians and researchers to accurately support individuals experiencing age-related changes in fluency performance.

## Supplementary Material

Supplementary_materials_kvag007

## Data Availability

Data is available at reasonable request to the first author.
