# Supplementary material for: The contribution of verbal delayed recall to phonemic but not semantic fluency in aging individuals
Source: Oxf Open Neurosci. 2026 Aug 4;5:kvag007. doi: 10.1093/oons/kvag007 (PMC13464686; doi:10.1093/oons/kvag007)
Supplement: Supplementary file 1 [file supplementary_materials_kvag007.zip › Electronic supplement_f.docx]

Electronic supplement for “**The contribution of verbal delayed recall to phonemic but not semantic fluency in aging individuals”** (Wehling, E.^1,2^, Lundervold, A.J.^1^, Wollschlaeger, D.^3^; ^1^ Department of Biological and Medical Psychology, University of Bergen, Norway; ^2^ Department of Neurology, Haukeland University Hospital Bergen, Norway; ^3^ Institute of Medical Biostatistics, Epidemiology and Informatics, University Medical Center of the Johannes Gutenberg-University Mainz, Mainz, Germany

**Supplementary Methods – Regression Diagnostics for GEE Poisson Models**

Outlier identification: Robust Mahalanobis distance flagged 7 participants as outliers with respect to the distribution of their covariate values. Visual inspection of individual records confirmed that all observed values were in the admissible range and plausible. Therefore, no participants were removed.

Supplementary Table 1: Covariate values for observations flagged as outliers based on robust Mahalanobis distance.

| Outlier ID | CWIT 3 | Digit Symbol | TMT B | WAIS vocabulary | CVLT ldfr |
| --- | --- | --- | --- | --- | --- |
| 1 | 69 | 49 | 145 | 51 | 9 |
| 2 | 63 | 29 | 170 | 57 | 12 |
| 3 | 106 | 45 | 84 | 61 | 11 |
| 4 | 56 | 44 | 123 | 40 | 7 |
| 5 | 67 | 40 | 174 | 43 | 7 |
| 6 | 93 | 37 | 190 | 66 | 15 |
| 7 | 116 | 39 | 115 | 58 | 14 |

Collinearity diagnostics: Variance inflation factors (VIF) for all covariates except for time ranged from 1.16 to 2.68, indicating minor collinearity. VIF for time in the course of the task was 130.50 due to its close but expected collinearity with pairwise interaction terms of cognitive performance measures and time.

Leverage and local influence: Diagnostic diagrams (Supplementary Figure 1-4) for assessing leverage of individual participants indicated no need to remove participants from the analysis data set.

Distribution of residuals: Plotting deviance residuals against predicted values (supplementary Figure 2, 4) did not show a systematic association that would have indicated model mis-specification.

Supplementary Table 2: Estimated coefficients (Relative Rates, “*RR*”) from separate GEE Poisson regression models for semantic fluency and phonemic fluency tasks based only on subjects without missing data. “*CI lo*” and “*CI up*” indicate 95 % confidence interval boundaries, “*p*” the corresponding p-value.

Supplementary Figure 1. Results from regression diagnostics for the GEE Poisson regression model for the phonemic (letter) fluency performance: DFBETAs values indicating the change in each coefficient estimate from omitting every participant separately from the model fitting.

Supplementary Figure 2: Results from regression diagnostics for the GEE Poisson regression model for the phonemic (letter) fluency performance: Leverage, Mahalanobis residuals, deviance residuals vs. fitted values as well as local influence of each participant.

Supplementary Figure 3: Results from regression diagnostics for the GEE Poisson regression model for the semantic (category) fluency performance: DFBETAs values indicating the change in each coefficient estimate from omitting every participant separately from the model fitting.

Supplementary Figure 4: Results from regression diagnostics for the GEE Poisson regression model for the semantic (category) fluency performance: Leverage, Mahalanobis residuals, deviance residuals vs. fitted values as well as local influence of each participant.
