# Supplementary material for: The contribution of verbal delayed recall to phonemic but not semantic fluency in aging individuals
Source: Oxf Open Neurosci. 2026 Aug 4;5:kvag007. doi: 10.1093/oons/kvag007 (PMC13464686; doi:10.1093/oons/kvag007)
Supplement: Supplementary file 1 [file supplementary_materials_kvag007.zip › Supplementary file for export.docx]

23-Apr-2026

Dear Dr. Wehling,

Thank you for submitting manuscript OXFNSC-2026-010, "Fluency tests and their associations with memory and executive functioning in aging individuals", to Oxford Open Neuroscience.  Your manuscript was seen by two reviewers and I have read it myself.

Both reviewers find this work of interest, but they also highlight important issues that require attention. I agree with this assessment, therefore I invite you to respond to the reviewer(s)' comments and revise your manuscript within the next 60 days from today date.

The two key areas noted by the reviewers are:

1) Issues surrounding statistical reporting (highlighted by reviewer 2). At the very least, you need to clarify and explain your statistical approach in more detail, clearly noting any potential caveats and limitations. The reviewer also suggests alternative analytic approaches. I encourage you to consider these approaches but I will not make this a formal condition for acceptance.

2) Interpretation of the role of episodic memory in fluency tasks (highlighted by both reviewers). For example (as noted by reviewer 1), just because CVLT scores are associated with fluency, does this implicate episodic memory itself or some other factor such as strategy selection?

Please also carefully consider all other points made by the reviewers.

Note that this journal operates with transparent peer review. This means that if your submission is accepted for publication, the full peer review history of your article will publish online alongside your article. This includes reviewer comments, editor decision letters, and your author responses.


To revise your manuscript, log into https://mc.manuscriptcentral.com/oxfnsc and enter your Author Centre, where you will find your manuscript title listed under "Manuscripts with Decisions."  Under "Actions," click on "Create a Revision."  Your manuscript number has been appended to denote a revision.

You may also click the below link to start the revision process (or continue the process if you have already started your revision) for your manuscript. If you use the below link you will not be required to login to ScholarOne Manuscripts.

*** PLEASE NOTE: This is a two-step process. After clicking on the link, you will be directed to a webpage to confirm. ***

https://mc.manuscriptcentral.com/oxfnsc?URL_MASK=58e20922075e4eec99c643b3e476b363

You will be unable to make your revisions on the originally submitted version of the manuscript.  Instead, revise your manuscript using a word processing program and save it on your computer.  Please also highlight the changes to your manuscript within the document by using the track changes mode in MS Word or by using bold or colored text.

<strong>REMINDER:</strong> if you have not already done so, when submitting your revised manuscript, please include a short alternative-text description under the figure legend for each figure. Alt-text is required to ensure the accessibility of our publications. Guidance is available on the journal’s Author Guidelines webpage and at <a href="https://academic.oup.com/pages/for-authors/journals/preparing-and-submitting-your-manuscript" target="_new">https://academic.oup.com/pages/for-authors/journals/preparing-and-submitting-your-manuscript</a>

Once the revised manuscript is prepared, you can upload it and submit it through your Author Centre.

When submitting your revised manuscript, you will be able to respond to the comments made by the reviewer(s) in the space provided.  You can use this space to document any changes you make to the original manuscript.  In order to expedite the processing of the revised manuscript, please be as specific as possible in your response to the reviewer(s).

IMPORTANT:  Your original files are available to you when you upload your revised manuscript.  Please delete any redundant files before completing the submission.

Because we are trying to facilitate timely publication of manuscripts submitted to the Oxford Open Neuroscience, your revised manuscript should be uploaded within the next 60 days from today date.  If it is not possible for you to submit your revision in a reasonable amount of time, we may have to consider your paper as a new submission.

Once again, thank you for submitting your manuscript to Oxford Open Neuroscience and I look forward to receiving your revision.

Sincerely,
Professor Sam Gilbert
Senior Editor, Oxford Open Neuroscience
sam.gilbert@ucl.ac.uk


Reviewer: 1

Comments to the Author
Summary:  
The study examines how verbal fluency (semantic and phonemic) relates to memory and executive functions in 132 healthy individuals aged 45-70 years. In addition to age, the study examines whether scores on several cognitive tests predict fluency performance. The investigation of temporal aspect of fluency tasks is interesting, but the study’s contribution to advance our understanding of aging-related processes or cognitive processes underlying verbal fluency is limited.  

- The manuscript should provide a theoretical rationale for conducting the study and explain its novelty.

- The literature review should include more recent empirical findings on the ognitive mechanisms underlying verbal retrieval in normal aging, in particular studies with larger samples such as Kljajevic, 2026 (N=1222)( https://psycnet.apa.org/record/2027-25961-001), Gonzalez-Burgos et al., 2019 (N = 446) (doi: 10.18632/aging.102040).

- The literature review should also distinguish between aging-related changes in semantic fluency and aging-related changes in phonemic fluency and discuss findings from previous studies.

p.6, line 10: How was it ensured that only healthy participants were included?

p. 7, line 10: Please provide more detail on how responses were recorded.

P. 7, line 44: The measure of cognitive flexibility used in the study is Trail Making Tests B. However, this test was previously criticized as a measure of cognitive flexibility, because of its high correlation with Trail A, which suggests that cognitive flexibility is not the primary component in this task (Lezak at al., 2004).

p. 8, line 48: Even though delayed free recall from word list learning tests such as CVLT are used as a measure of episodic memory, it strongly depends on search strategies, which are supported by executive functions (EFs). At best delayed recall from CVLT measures retention of decontextualized verbal material, not recollection of an episode. (Episodic memories are not only about ‘what; but also about ‘where’ and ‘how’.) Since people often use strategies to group words in encoding and retrieval, scores on CVLT and similar tests reflect semantic memory and use of strategies rather than a past event. In addition, encoding, storage, and retrieval of lists of words require language abilities and attention, meaning that suboptimal performance on the delay task may reflect difficulties in these other abilities while episodic memory could be intact. So, while delayed recall can be related to episodic memory, it is better understood as a composite measure (blending retrieval strategy, semantic organization, language ability, attention) rather than a measure of episodic memory. Splitting a 60 second fluency task into four 15 sec bins does not turn it into an episodic event.

p. 9., line 3: The manuscript would benefit from elaborating on how this analysis was performed, e.g. how were words separated into 15 sec blocks for instance in cases where they were stretched across the block’s border, what program was used etc.

p.13, line 48: Please clarify/rephrase a ‘wider age range’: the study by Stolwyk et al. referred here included persons between 65 and 87 years of age (range = 22 vs. 25 age range in the present study).  

p. 15, line 25: Please explain the relevance of orthographic network for the present task, i.e. how were word lists presented?

p. 15, lines 37-38: the conclusion that episodic memory facilitates retrieval is not supported by this association. Both tasks , phonemic fluency and delayed recall, involve multiple processes so if the two tasks correlate it does not necessarily mean that episodic memory is the shared underlying mechanism, because it could be that some people are better at retrieving and organizing verbal information.

- The limitations of the study are not discussed, except for a very brief mention of one issue at the end of the manuscript.

- The Discussion ends abruptly.

- The Conclusion is too short and doesn’t highlight the study’s contribution in the context of other recent developments or presents implications of its finding for the field of cognitive aging.


Reviewer: 2

Comments to the Author
This manuscript examines associations between phonemic and semantic verbal fluency and several cognitive measures, including executive functioning, processing speed, vocabulary, and episodic memory, in 132 middle-aged to older individuals. The topic is relevant, and the time-course approach is potentially useful, but the current version overstates the evidence for episodic memory and does not describe the modeling strategy clearly enough for the main claims to be fully convincing.

My biggest concern concerns the statistical analysis, including model specification, model assessment, interpretation, and the gap between the strength of the claims and the actual statistical support presented. I was also disappointed to see a manuscript submitted to an open neuroscience journal without links to either the data or an analysis repository, which makes the already poorly specified statistical analysis section impossible to replicate.

Major comments
1. Statistical model needs revision. The authors state that they used Poisson regression with generalized estimating equations for repeated measures across four 15-second intervals, but the model description remains too unclear for readers to understand exactly what was analyzed. I strongly recommend replacing the current approach with a generalized linear mixed model using a Poisson link, or a negative binomial model if overdispersion is present, so that the repeated-measures structure is modeled directly at the participant level across all time bins. At minimum, the model should include participant-specific random intercepts and a clearly specified time effect. A by-participant random slope for time should also be tested. Model comparison should be used to justify the random-effects structure, overdispersion should be assessed, and model diagnostics should be reported.
2. The outcome definition is internally inconsistent. The Methods state that each participant contributed four repeated observations, one for each 15-second interval, but the manuscript also says that fluency was modeled as “rates of produced words per minute” using total words divided by 3 for phonemic fluency and by 2 for semantic fluency. These are not the same outcome definitions. The authors should state explicitly whether the dependent variable was the count of correct words in each 15-second bin, a rate with an offset, or an aggregate task score.
3. The handling of time is unclear and confusing. The manuscript says that “time period” was entered as a continuous predictor, but the Results later refer to a “late interval,” and the text interprets effects as if they were specific to later task phases. This is ambiguous and makes it unclear what the reported “time effect” actually refers to. The authors should model and report time bins explicitly, ideally as a factor, so readers can see whether effects differ across all four intervals rather than being reduced to an unclear linear trend. This is especially important because the main novelty claim concerns episodic memory emerging later in phonemic fluency.
4. Relatedly, Figure 4 is difficult to understand. It is completely unclear to me how the interaction figure was derived if, according to Table 4, the interaction only involved counts for the late time interval.
5. Predictor selection is not justified. Correlation is not a valid basis for predictor selection, because bivariate screening is not a principled approach to multivariable model construction. Instead, I would recommend first using an exploratory factor analysis or principal component analysis to assess whether there are common underlying cognitive components that can then be entered as predictors into the model. It is clear that correlations between several neuropsychological assessments are high (see Table 3; for example, processing speed/DSST with other tests). I was also not convinced by the labels assigned to the tests. If each cognitive function is represented by only one test, that is a weak operationalization and needs to be examined more critically. At present, I cannot trust the model results because of likely substantial collinearity among predictors.
6. Why was only sex retained from the demographic variables in the regression models? Age was a core theoretical variable, and excluding it from the models because of a non-significant bivariate correlation is not convincing. Age is central to the paper’s framing and should be included in the main models, especially because the age range is very broad and will most likely matter. A 45-year-old and a 77-year-old should not simply be treated as equivalent members of a single “aging” sample.
7. Were fluency task order and category/letter order fixed or counterbalanced?
8. The semantic outcome needs stronger justification. Please justify combining “animals” and “boys’ names” into one semantic outcome. Two categories are already a very small basis to begin with, and names are a particularly unusual category that is strongly influenced by culture and cohort.
9. The central claim is stronger than the data justify. In Table 4, the “episodic memory × late interval” effect for phonemic fluency is statistically significant, but the reported RR is 1.000 with CI 1.000–1.000, which makes the effect size practically uninterpretable. The manuscript should report a scaled effect, marginal predictions, or simple slopes that show the actual magnitude.
10. Several statements are inconsistent with the tables. Table 2 lists similar total raw word counts for phonemic and semantic fluency because phonemic totals aggregate three letters whereas semantic totals aggregate two categories, yet the text alternates between totals and per-minute rates in ways that are easy to misread. Similarly, it remained unclear which final outcome metric was actually used in the statistical models: counts per time bin, rates divided by 2/3, or total counts.
11. There was substantial imputation due to missing data. Using a mixed-effects model would also help here, since missing repeated observations are less problematic in that framework. If imputation is still necessary, it should be stated clearly how it was done and how it affected the analysis.
12. The reported evidence for the role of episodic memory in verbal fluency is weak and strongly overstated in the title and Discussion. The effect is emphasized mainly for phonemic fluency over time, and it does not clearly generalize to semantic fluency. The current result does not support a broad clinical conclusion that episodic memory should routinely be considered when interpreting fluency performance. The authors should narrow the claim to what was actually shown in the fitted models.
13. The Methods section is badly structured. First, a general cognitive assessment is described, and then each test is described again in detail afterward. It is unclear why the section is organized this way. Also, under “Cognitive assessment,” the authors write “the overall level of cognitive recovery of each group.” Why “recovery”? I understood these to be healthy individuals.

Minor comments
1. The TMT is coded such that higher numbers indicate worse performance, whereas for most other tests higher scores indicate better performance. Was this handled appropriately?
2. p. 5, l. 17: I believe a “not” is missing.
3. p. 8, l. 11: The test is called “WAIS,” not “WASI.”
4. p. 16, l. 13: The year is missing for the reference “Greenberg et al.”

Associate Editor
Comments to the Author:
(There are no comments.)

Dear Professor Gilbert,
Thank you for valuable comments from reviewers and editor and the opportunity to revise the manuscript "Fluency tests and their associations with memory and executive functioning in aging individuals" which title was changed according to Reviewer 2’s recommendations to “The contribution of delayed recall to phonemic but not semantic fluency in aging individuals”.
Regarding the two major issues you highlighted, we have discussed them carefully with all authors and made revisions which we find improved the clarity of the manuscript. We would like to thank the reviewers for doing an thoroughly job by pointing them out. Please find the detailed answers to the reviewers below. Due to the extensive revisions made across large parts of the manuscript, we here provide only the rationale and page numbers for some points in order to maintain the readability of the responses. Changes to the manuscript are highlighted in yellow face.
We hope that the manuscript can now be considered for publication.
On behalf of all authors,
Sincerely,
Eike Wehling

Detailed answers to reviewer 1:
-The manuscript should provide a theoretical rationale for conducting the study and explain its novelty.
• We agree that the rationale and novelty needed more consideration in the introduction. We have discussed this issue and made considerable changes. The rational and novelty are now included throughout the text in the introduction (including Page 2, line 19 ff, page 4 line 5 ff, page 5/6 line 24 ff and page 6 line 16 ff).
- The literature review should include more recent empirical findings on the cognitive mechanisms underlying verbal retrieval in normal aging, in particular studies with larger samples such as Kljajevic, 2026 (N=1222)( https://psycnet.apa.org/record/2027-25961-001), Gonzalez-Burgos et al., 2019 (N = 446) (doi: 10.18632/aging.102040).
- The literature review should also distinguish between aging-related changes in semantic fluency and aging-related changes in phonemic fluency and discuss findings from previous studies.
• Thank you for pointing out important findings by other authors which we included in the manuscript. Additionally, we included a paragraph on findings regarding cognitive measures and their association in aging individuals.
• Page 5 line 11 ff
p.7, line 18 ff: How was it ensured that only healthy participants were included?
• We deleted the word “healthy” and included a sentence regarding mild cognitive impairment. This now reads:
• In total, this study included n = 132 community-dwelling aging individuals (see Table 1 for sample characteristics). To control for potential mild cognitive impairment, we verified that none of the participants scored 1.5 SD below age matched normative data on the CVLT delayed recall, a commonly used cutoff for mild cognitive impairment (Petersen, 2004).

p. 8, line 19: Please provide more detail on how responses were recorded.
• We added a sentence to clarify this issue:
• All words (correct answers, repetitions and set loss errors) were recorded in writing using designated sheets within 15 second intervals. Words generated while crossing the border between intervals were attributed to the earlier interval.
p. 9, line 9: The measure of cognitive flexibility used in the study is Trail Making Tests B. However, this test was previously criticized as a measure of cognitive flexibility, because of its high correlation with Trail A, which suggests that cognitive flexibility is not the primary component in this task (Lezak at al., 2004).
• We address this issue and have added a phrase:
• Although the Trail Making Test Part B (TMT-B) is commonly viewed as a measure of cognitive flexibility, some authors argue that flexibility may not be the primary contributing factor (Lezak et al., 2012).
p. 6, line 1: Even though delayed free recall from word list learning tests such as CVLT are used as a measure of episodic memory, it strongly depends on search strategies, which are supported by executive functions (EFs). At best delayed recall from CVLT measures retention of decontextualized verbal material, not recollection of an episode. (Episodic memories are not only about ‘what; but also about ‘where’ and ‘how’.) Since people often use strategies to group words in encoding and retrieval, scores on CVLT and similar tests reflect semantic memory and use of strategies rather than a past event. In addition, encoding, storage, and retrieval of lists of words require language abilities and attention, meaning that suboptimal performance on the delay task may reflect difficulties in these other abilities while episodic memory could be intact. So, while delayed recall can be related to episodic memory, it is better understood as a composite measure (blending retrieval strategy, semantic organization, language ability, attention) rather than a measure of episodic memory. Splitting a 60 second fluency task into four 15 sec bins does not turn it into an episodic event.

• Reviewer 1 points out a well-justified issue, and we are aware that delayed recall as used in our study lacks the spatiotemporal contextual binding that characterizes episodic recollection. We have therefore changed the entire manuscript using “delayed recall” referring to the memory measure we used and introduced this aspect in the introduction (page 6 line 1 ff).
• We also appreciate the Reviewer point on the splitting of the fluency task, which may have been unclear. We do not regard the temporal segmentation of the fluency task as creating or measuring episodic events. The 15-second bins are intended to characterize the time course of the fluency task. We made considerable changes in the methods section (in particular in the section Statistical analyses page 10) to make this aspect clearer.
p. 8., line 20: The manuscript would benefit from elaborating on how this analysis was performed, e.g. how were words separated into 15 sec blocks for instance in cases where they were stretched across the block’s border, what program was used etc.
• For clarification of this issue, we included the following sentence:
• “Words generated while crossing the border between intervals were attributed to the earlier interval.”
p.17, line 5 ff: Please clarify/rephrase a ‘wider age range’: the study by Stolwyk et al. referred here included persons between 65 and 87 years of age (range = 22 vs. 25 age range in the present study).
• The paragraph was changed to increase clarity. This now reads:
• Bivariate correlations showed no association of verbal fluency performance with age, in contrast to previous studies (Bryan et al., 1997; Henry & Phillips, 2006; Rodriguez-Aranda & Martinussen, 2006 but see also Sauzeon et al., 2010; Shao, Janse, Visser, & Meyer, 2014). Varying findings may be due to the included age-ranges in the studies. We focused specifically on aging individuals to be able to interpret the performance patterns of this demographically growing group (Shao et al., 2014; Stolwyk et al., 2015).
p. 15, line 25: Please explain the relevance of orthographic network for the present task, i.e. how were word lists presented?
• This paragraph was rephrased and the comment on the orthographic network is removed.
p. 19, line 7 ff: the conclusion that episodic memory facilitates retrieval is not supported by this association. Both tasks, phonemic fluency and delayed recall, involve multiple processes so if the two tasks correlate it does not necessarily mean that episodic memory is the shared underlying mechanism, because it could be that some people are better at retrieving and organizing verbal information.
• The complexity of both verbal fluency and delayed recall is now addressed throughout the manuscript and at suggestion that these two measures require strategic search in long-term memory is discussed starting on page 19 line 7
p. 21 The limitations of the study are not discussed, except for a very brief mention of one issue at the end of the manuscript.
• A paragraph on the limitations and strengths of the study is now included.
• Page 21
-The Discussion ends abruptly.
• The discussion has been revised.
• Pages 18-21
-The Conclusion is too short and doesn’t highlight the study’s contribution in the context of other recent developments or presents implications of its finding for the field of cognitive aging.

• The conclusion has been revised and contains now the studies’ contribution and implications of the findings. Page 22:
• Including both phonemic and semantic fluency, the current study reveals that word knowledge, inhibition and delayed recall contribute to phonemic fluency performance while shifting ability contributed to semantic fluency performance in a group of aging individuals. These findings contribute to novel insight by examining whether cognitive measures interact with performance across different parts of the test, which was shown for delayed recall in phonemic fluency. A better understanding of fluency measures is essential for clinicians and researchers to accurately support individuals experiencing age-related changes in fluency performance.
Reviewer 2
Major comments
1. Statistical model needs revision. The authors state that they used Poisson regression with generalized estimating equations for repeated measures across four 15-second intervals, but the model description remains too unclear for readers to understand exactly what was analyzed. I strongly recommend replacing the current approach with a generalized linear mixed model using a Poisson link, or a negative binomial model if overdispersion is present, so that the repeated-measures structure is modeled directly at the participant level across all time bins. At minimum, the model should include participant-specific random intercepts and a clearly specified time effect. A by-participant random slope for time should also be tested. Model comparison should be used to justify the random-effects structure, overdispersion should be assessed, and model diagnostics should be reported.
• Generalized Estimating Equations vs. Generalized Linear Mixed Model: The GEE model uses a robust sandwich estimator for the covariance matrix (DOI 10.12691/ajams-3-6-5, 10.18637/jss.v015.i02) which makes it appropriate for potentially overdispersed count data. As noted in DOI 10.1080/02664763.2020.1788518, Poisson GEE models may indeed outperform GLMMs for this application. Furthermore, this publication comments on convergence issues that may render fitting GLMMs difficult. It is also our experience that GLMMs, especially those with complex random-effects structure as suggested by Reviewer 2, can be difficult to fit, in particular with limited sample size as in our study. Moreover, our goal was to characterize marginal, i.e., population-level associations which is more naturally captured by a GEE model as opposed to the subject-specific (conditional) GLMM model required to estimate inter-individual variance. Therefore, we opted to keep the GEE model approach.
• Unclear model description: We agree that the model description was hard to follow, and have now substantially revised this section.
• Model diagnostics: We now provide model diagnostics in an electronic supplement.
• Changes pages 10-13
2. The outcome definition is internally inconsistent. The Methods state that each participant contributed four repeated observations, one for each 15-second interval, but the manuscript also says that fluency was modeled as “rates of produced words per minute” using total words divided by 3 for phonemic fluency and by 2 for semantic fluency. These are not the same outcome definitions. The authors should state explicitly whether the dependent variable was the count of correct words in each 15-second bin, a rate with an offset, or an aggregate task score.
• We agree that the description of test and analysis methods were not sufficiently clear. We have now substantially revised the description of how the outcome was calculated. The fluency performance values used in the Poisson regression models were absolute counts, and by using the appropriate offset in the models, results are expressed as words-per-minute rates.
• Changes pages 10-13
3. The handling of time is unclear and confusing. The manuscript says that “time period” was entered as a continuous predictor, but the Results later refer to a “late interval,” and the text interprets effects as if they were specific to later task phases. This is ambiguous and makes it unclear what the reported “time effect” actually refers to. The authors should model and report time bins explicitly, ideally as a factor, so readers can see whether effects differ across all four intervals rather than being reduced to an unclear linear trend. This is especially important because the main novelty claim concerns episodic memory emerging later in phonemic fluency.
• We agree that the description of methods with respect to handling the time intervals was unclear. We have now substantially revised the Statistics section (page 10-12) to indicate how time was included as a continuous variable.
• With respect to treating the time interval as a categorical variable vs. using time as a continuous variable, we prefer to keep time as continuous in the model for the following reasons: Based on theoretical grounds, it is to be expected that performance drops over time in a monotonic fashion. A log-linear relationship (= exponential dropoff) captures this assumption and may serve as a realistic approximation over the short time span observed (1 minute), in particular considering the descriptive trend visible in Figure 1. Using interval as a categorical variable would not reflect the monotonicity restriction. Moreover, modeling a log-linear relationship is more parsimonious with only 1 parameter as opposed to 3 parameters for the categorical variable and thus decreases uncertainty and improves power.
• Changes pages 10-13
4. Relatedly, Figure 4 is difficult to understand. It is completely unclear to me how the interaction figure was derived if, according to Table 4, the interaction only involved counts for the late time interval.
• We assume that Reviewer 2 here refers to Figure 2. By revising the statistics section, we hope that it is now clear that the pairwise interaction terms between time course during each task and each cognitive performance covariate were entered into the models. Reviewer 2 correctly points out that the manuscript contained no description of the estimated marginal means method used to derive these predictions. We have added this description to the Statistics section and to the caption of Figure 2.
5. Predictor selection is not justified. Correlation is not a valid basis for predictor selection, because bivariate screening is not a principled approach to multivariable model construction. Instead, I would recommend first using an exploratory factor analysis or principal component analysis to assess whether there are common underlying cognitive components that can then be entered as predictors into the model. It is clear that correlations between several neuropsychological assessments are high (see Table 3; for example, processing speed/DSST with other tests). I was also not convinced by the labels assigned to the tests. If each cognitive function is represented by only one test, that is a weak operationalization and needs to be examined more critically. At present, I cannot trust the model results because of likely substantial collinearity among predictors.
• We agree that covariate selection is a crucial step in model building. Indeed, covariate selection was based on theoretical grounds except for education and vocabulary. Here, their high correlation motivated us to drop one of the two variables to avoid collinearity issues. We chose to retain only vocabulary as the more direct measure of specific neuropsychological performance. Apart from this specific decision, bivariate correlations did not inform covariate selection.
• Cognitive performance measures were assessed using standardized tests with known reliability and validity characteristics. These tests are used both in scientific evaluations as well as in clinical practice. Using PCA or EFA as a data reduction technique would create artificial new variables that cannot be meaningfully linked to previous studies or clinical practice. Furthermore, the results from the PCA or EFA would be based on our sample characteristics, their reliability and validity would likely not reach those from standardized tests.
• Collinearity: We now provide collinearity diagnostics in the electronic supplement.
6. Why was only sex retained from the demographic variables in the regression models? Age was a core theoretical variable, and excluding it from the models because of a non-significant bivariate correlation is not convincing. Age is central to the paper’s framing and should be included in the main models, especially because the age range is very broad and will most likely matter. A 45-year-old and a 77-year-old should not simply be treated as equivalent members of a single “aging” sample.
• We regret that the methods section was unclear with respect to the process of covariate selection. Bivariate correlations did not inform the decision to not include age as a predictor. We have revised the Statistics section to explain our rationale for not including age as a predictor (page 12 line 11 ff). Specifically, we assume that any effect of age on verbal fluency performance is mediated by the specific cognitive performance measures which are included in the model.
7. Were fluency task order and category/letter order fixed or counterbalanced?
• Administration followed the manual, starting always with phonemic fluency. This is now clarified (page 8 line 14)
• All participants first performed the phonemic fluency subtests, followed by the semantic fluency subtests according to the manual.
8. The semantic outcome needs stronger justification. Please justify combining “animals” and “boys’ names” into one semantic outcome. Two categories are already a very small basis to begin with, and names are a particularly unusual category that is strongly influenced by culture and cohort.
• The combination of the two semantic categories was based on the standardized test of semantic fluency provided in the D-KEFS manual which is one of the most frequently used measures in countries using the D-KEFS battery (North America and several states in Europe).
• We are aware that there can be cultural/language differences regarding the number of words produced as shown by doi: 10.1080/02699200902839800. These authors argue that culture may be reflected in qualitative aspects such as organization of types of words (prototypes) semantic categories and qualitative differences such as word length. This aspect is now addressed in the limitations section.
• Changes pages 21-22
9. The central claim is stronger than the data justify. In Table 4, the “episodic memory × late interval” effect for phonemic fluency is statistically significant, but the reported RR is 1.000 with CI 1.000–1.000, which makes the effect size practically uninterpretable. The manuscript should report a scaled effect, marginal predictions, or simple slopes that show the actual magnitude.
• We agree that interpretation of the interaction coefficients is made difficult by the fine resolution of the time scale (seconds) and episodic memory (words), leading to numerically very small estimates. We now also report results as a rate ratio for 5 words in the episodic memory task at 10 seconds and at 20 second in the course of the phonemic fluency performance task (page 16 line 2 and line 15). Furthermore, marginal predictions are shown in Figure 2.
10. Several statements are inconsistent with the tables. Table 2 lists similar total raw word counts for phonemic and semantic fluency because phonemic totals aggregate three letters whereas semantic totals aggregate two categories, yet the text alternates between totals and per-minute rates in ways that are easy to misread. Similarly, it remained unclear which final outcome metric was actually used in the statistical models: counts per time bin, rates divided by 2/3, or total counts.
• We agree that reporting was inconsistent. We have revised the description of methods and results accordingly
• Changes pages 11 line 25/12 line 1 ff
11. There was substantial imputation due to missing data. Using a mixed-effects model would also help here, since missing repeated observations are less problematic in that framework. If imputation is still necessary, it should be stated clearly how it was done and how it affected the analysis.
• Missing data was mainly the result from not being able to assess shifting performance in the Trail Making Test for 15 participants due to an organizational issue. As this is a static variable without repeated measures, imputation is necessary regardless of statistical model to avoid the risk of selection bias from dropping these participants. We revised reporting of the imputation strategy (page 12 line 19) and included the results from the complete case analysis as a sensitivity analysis in the electronic supplement.
12. The reported evidence for the role of episodic memory in verbal fluency is weak and strongly overstated in the title and Discussion. The effect is emphasized mainly for phonemic fluency over time, and it does not clearly generalize to semantic fluency. The current result does not support a broad clinical conclusion that episodic memory should routinely be considered when interpreting fluency performance. The authors should narrow the claim to what was actually shown in the fitted models.
• We agree that that the conclusion was too broad and have now rephrased the discussion (page 19-20) as well as the conclusion (page 22)
• We have changed the title of the paper as well as large parts of the discussion. The new title is
• “The contribution of verbal delayed recall to phonemic but not semantic fluency in aging individuals”
• Changes pages 19-20 and page 22

13. The Methods section is badly structured. First, a general cognitive assessment is described, and then each test is described again in detail afterward. It is unclear why the section is organized this way. Also, under “Cognitive assessment,” the authors write “the overall level of cognitive recovery of each group.” Why “recovery”? I understood these to be healthy individuals.
• We have revised the method section and removed the word “recovery” which was a flaw. This now reads (page 4 line 4):
• Participants were assessed with tests described below. To provide an indication of performance levels, standardized scores were computed for each measure according to normative data from the respective test manuals (Table 1).

Minor comments
1. The TMT is coded such that higher numbers indicate worse performance, whereas for most other tests higher scores indicate better performance. Was this handled appropriately?
• The TMT scores indicate seconds to perform the task, so that higher scores indicate longer time to finish. To clarify this issue, we included a sentence in the test description:
• Since for both the CWIT and the TMT-B tests scores represent the time elapsed to complete the task, higher numbers indicate lower performance on the task.
• Changes page 9 line 11


2. p. 5, l. 17: I believe a “not” is missing.
• The paragraph was rephrased. This now reads:
• Moreover, we expect significant associations between word knowledge, executive function, and processing speed and phonemic fluency whereas semantic fluency is expected to be associated with word knowledge and executive function.
• Changes page 7 line 7
3. p. 8, l. 11: The test is called “WAIS,” not “WASI.”
• We used the subtest of the Wechsler Abbreviated Intelligence Scale which is a short form of the WAIS. This is now revised in the text.
• Changes page 10 line 1
4. p. 16, l. 13: The year is missing for the reference “Greenberg et al.”
• Since the discussion was changed this reference is not included anymore.
